# Breakthrough Acute Necrotizing Invasive Fungal Rhinosinusitis by *Alternaria* *alternata* in a Patient with Acute Lymphoblastic Leukemia on Anidulafungin Therapy and Case-Based Literature Review

**DOI:** 10.3390/jof8080879

**Published:** 2022-08-20

**Authors:** Giorgos Tyrellis, Maria Siopi, Danai Leventakou, Alexander Delides, Pavlos Maragkoudakis, George Korres, Christina Apostolopoulou, Alina-Roxani Gouloumi, Vasiliki Pappa, Spyros Pournaras, Ioannis Panayiotides, Joseph Meletiadis

**Affiliations:** 1 2nd Otolaryngology Department, Attikon University General Hospital, Medical School, National and Kapodistrian University of Athens, 12462 Athens, Greece; 2 Clinical Microbiology Laboratory, Attikon University General Hospital, Medical School, National and Kapodistrian University of Athens, 12462 Athens, Greece; 3 2nd Department of Pathology, Attikon University General Hospital, Medical School, National and Kapodistrian University of Athens, 12462 Athens, Greece; 4 2nd Department of Internal Medicine, Hematology Unit, Attikon University General Hospital, Medical School, National and Kapodistrian University of Athens, 12462 Athens, Greece

**Keywords:** invasive necrotizing rhinosinusitis, *Alternaria* spp., breakthrough

## Abstract

*Alternaria* spp. have emerged as opportunistic pathogens particularly in immunosuppressed patients. A case of a breakthrough acute invasive fungal rhinosinusitis (AIFRS), caused by *Alternaria alternata*, is reported in a patient with acute lymphoblastic leukemia (ALL) on anidulafungin therapy, who was successfully treated with liposomal amphotericin B and surgical intervention. To date, 20 cases of AIFRS due to *Alternaria* spp. have been described, 19 in the USA and 1 in Chile, making this case report the first case of AIFRS due to *Alternaria* in Europe. The patients had median (range) age 25 (2–56) years (65% female), almost all of them (19/20) with hematological diseases and severe neutropenia (8–41 days pre-infection). Amphotericin B was the most frequently used antifungal agent, either alone or in combination. In all of the cases, systemic antifungal therapy was combined with surgery. Despite stabilization or improvement of the AIFRS, mortality was 38% (5 days to 8 months post-surgical debridement) due to their underlying disease or other infections without sign of AIFRS at autopsy.

## 1. Introduction

Fungal rhinosinusitis (FRS) comprises a wide spectrum of diseases, ranging from superficial colonization, allergic manifestations, to life-threatening invasive diseases. Given that the presenting clinical signs and radiographic features are often non-specific, FRS diagnosis poses a challenge and its establishment requires histopathologic evaluation and culture of the nasal biopsy material [1]. The invasive form of FRS is further categorized to acute, chronic and granulomatous, depending on the time course of infection, the host factors and histopathologic characteristics [2]. Acute invasive FRS (AIFRS) is a relatively rare but lethal disease, particularly in patients with hematological malignancies and low absolute neutrophil counts [1]. It is a rapidly progressive infection characterized by submucosal infiltration of fungal pathogens from the nasal cavity or paranasal sinuses into the adjacent orbits, as well as the brain parenchyma and vascular cavernous sinus pathways [3]. Considering its aggressive clinical course (<4 weeks), the prompt surgical debridement in conjunction with an appropriate antifungal therapy and reversal/reduction in the underlying causative immunosuppression state are the mainstays of its management [4]. AIFRS is largely attributable to *Aspergillus* and Mucorales [1]; albeit other atypical fungal pathogens, such as *Fusarium* spp. [5,6,7] and dematiaceous fungi [6,7,8], have been rarely described as disease-etiological agents and have been associated with worse survival [6]. We herein report the first case in Europe of acute necrotizing invasive FRS caused by *Alternaria alternata* in a patient with acute lymphoblastic leukemia (ALL).

## 2. Case Report

**Figure 1:**
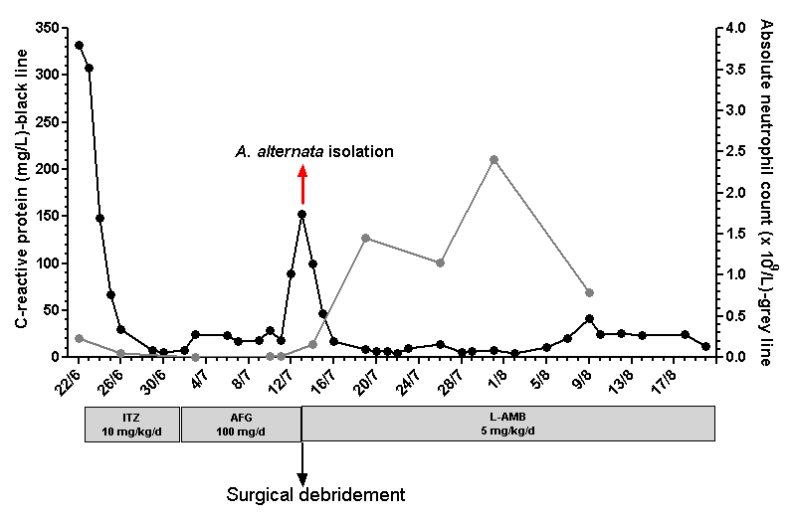
C-reactive protein (CRP) levels, absolute neutrophil count and antifungal therapy on a daily basis. Significant CRP reduction was observed after surgical intervention and introduction of liposomal amphotericin B (L-AMB) as antifungal therapy (13/7) (ITZ: itraconazole; AFG: anidulafungin).

A 36-year-old female farmer living in the countryside with a past medical history of hypothyroidism presented to the emergency department with difficulty in swallowing and bilateral cervical lymphadenopathy. An otolaryngology (ENT) examination was consulted and endoscopy revealed an enlargement of the Waldeyer’s ring lymphoid tissue, which was subsequently confirmed by computed tomography (CT) scan findings. The blood tests demonstrated leukocytosis (white blood cells count 24.8 × 10^9^/L), severe neutropenia (absolute neutrophil count 0.23 × 10^9^/L) and elevated C-reactive protein (CRP) level (332 mg/L), while the flow cytometric analysis of the bone marrow biopsy was indicative of ALL (Table 1). The patient was admitted to the hematology unit for further treatment by aggressive chemotherapy (Hyper-CVAD). During her hospitalization, she received i.v. antibiotic (metronidazole 500 mg q8h for 8 days, piperacillin-tazobactam 4.5 g q6h for 8 days) and antifungal (itraconazole 5 mg/kg q12h for 6 days) therapy. After six days of antifungal treatment, the patient complained about dizziness and nausea, and thus the attending hematologist decided to replace itraconazole with anidulafungin (a single 200 mg loading dose, followed by 100 mg q24h). Two days after the change, she developed fever and the antibacterial drugs were replaced by meropenem (2 g q8h) and vancomycin (1 g q12h). At that day, the CRP level was 8 mg/L and in the following 10 days, the median (range) CRP concentration was 21 (17–28) mg/L (Figure 1).

**Figure 2:**
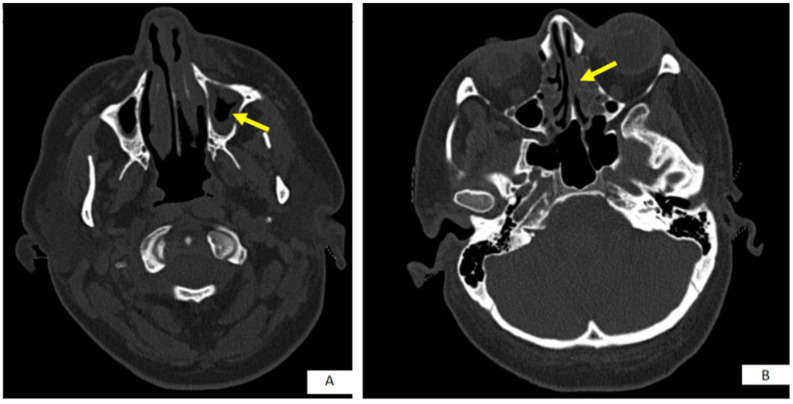
Axial computed tomography of nose and paranasal sinuses shows opacification of (**A**) the left maxillary sinus (yellow arrow); and (**B**) the left ethmoidal air cells (yellow arrow).

On the 18th day of antifungal treatment (12th on anidulafungin therapy), she complained of acute facial pain, nasal obstruction and left eye lacrimation, while her laboratory results showed elevated CRP (89 mg/L). A computed tomography (CT) scan revealed opacification of the left ethmoid air cells and maxillary sinus (Figure 2).

**Figure 3:**
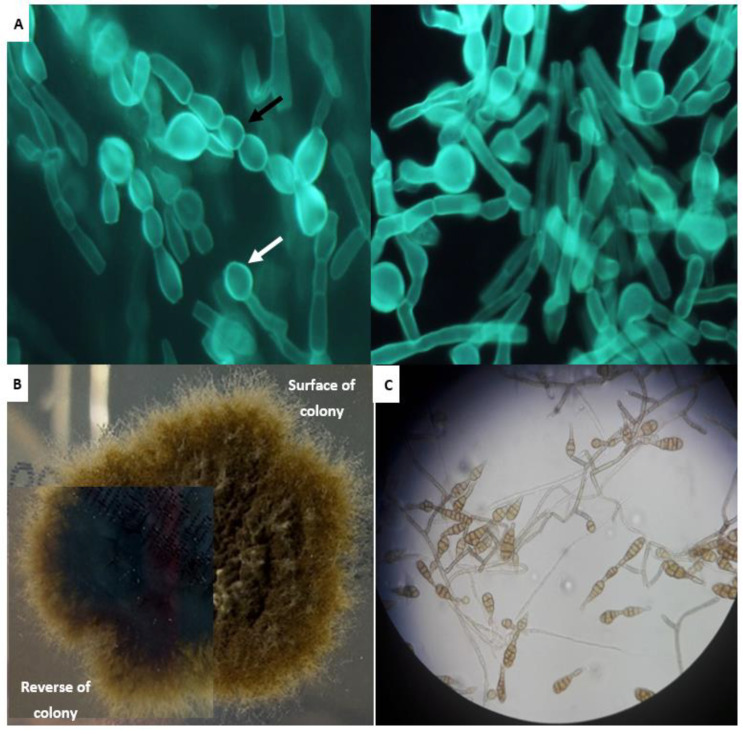
(**A**) Tissue direct fluorescence microscopy using Blankophor P revealed the presence of hyphae with closely spaced, constricted septations producing a “string of beads” appearance (black arrow) and large, thick-walled vesicular swellings resembling chlamydoconidia at their ends (white arrow) (original magnification 400×); (**B**) Powdery, olivaceous colony with brownish-black pigmentation grown on Sabouraud dextrose agar after five days of incubation at 30 °C; (**C**) Medium-brown, muriform conidia with transverse and longitudinal septations and a short, apical terminal beak (original magnification 400×).

**Figure 4:**
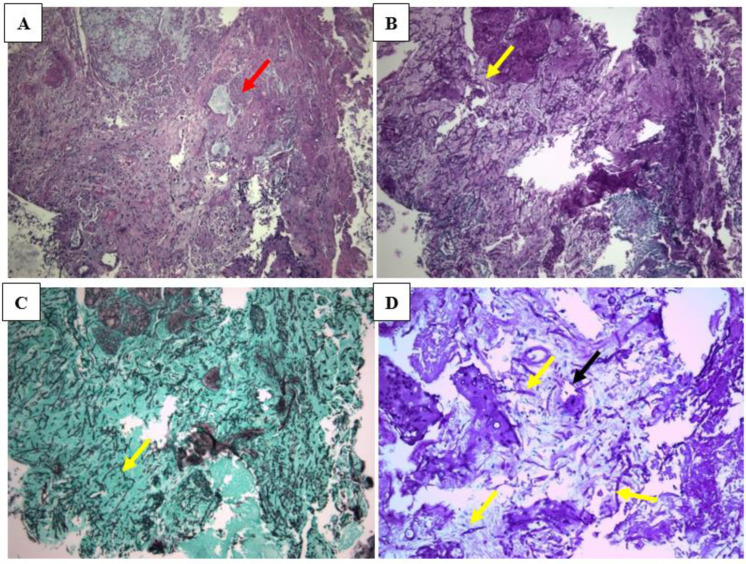
Nasal vestibular mucosa partially covered by squamous epithelium, with extensive necrosis (red arrow) (**A**); many septate fungal hyphae branching at right angles were seen both with PAS (**B**) and Grocott’s silver impregnation (**C**) stains. Hyphae were found within thrombotic small vessels (**D**; center). (**A**) Hematoxylin and Eosin, ×10; (**B**) PAS, ×10; (**C**) Grocott’s silver impregnation, ×10; (**D**) PAS, ×20: yellow arrows pointing to hyphae, black arrow pointing to hypha partially within vessel wall.

An ENT endoscopy showed white and blanched areas of the mucosa of the nasal vestibule and tissue with necrotizing sections. The tissue samples were sent for both histological and microbiological examination. Direct examination with Blankophor P fluorescent staining revealed the presence of septate branching hyphae, having mostly closely spaced, constricted septations producing a “string of beads” appearance, and large, thick-walled vesicular swellings resembling chlamydoconidia at the ends of the hyphae (Figure 3A). The histology showed fragments of nasal mucosa with extensive infarction-like necrosis, as well as severe chronic inflammation. The necrotic areas contained many septate fungal hyphae branching at right angles; moreover, the hyphae were seen within the vessels (Figure 4). It was then decided to replace anidulafungin with liposomal amphotericin B (5 mg/kg/day).

Given the aforementioned findings, on the same day the patient underwent urgent surgical debridement with endoscopic sinus surgery that included medial maxillary antrostomy, ethmoidectomy and resection of part of the nasal septum (pre-surgery CRP 152 mg/L, Figure 1). The post-debridement tissues showed the same histologic picture as previously described, albeit with very few hyphae; and culture based on the results obtained earlier that day. Her postoperative course was uneventful and the symptoms resolved immediately. Two days post-surgery, the CRP concentration showed a remarkable reduction (47 mg/L, Figure 1) and the ENT endoscopy revealed a distinguished improvement through the extinction of the blanched areas observed during the endoscopy prior to the surgery. The antifungal treatment with liposomal amphotericin B was continued for 38 days, while the patient was advised to use a mupirocin topical cream two times/day and to perform saline (0.9%) nasal irrigation three times/day for 2 weeks. Since then, she underwent follow-up CT scan and ENT examination for 6 months, without any pathological findings.

## 3. Mycological Workup

### 3.1. Culture

The tissue samples removed during the ΕΝΤ endoscopy were inoculated onto blood agar, MacConkey agar, chocolate agar and thioglycolate broth (Oxoid) incubated at 37 °C, as well as Sabouraud dextrose (SAB) agar with gentamicin and chloramphenicol (Oxoid) and 10 mL liquid SAB medium (Oxoid) with 0.05 g/L chloramphenicol (Applichem) incubated at 30 °C. Grayish white wooly colonies appeared within 2 days of inoculation in SAB, which became dark olive-green and with brownish-black pigment on further incubation (Figure 3B). The microscopy revealed branches of septate brownish hyphae with multiple chains of large, medium-brown, muriform conidia with transverse and longitudinal septations, whereof some had a short, apical beak at their distal end (Figure 3C). The isolate was identified as *Alternaria* spp. and was subjected to confirmatory molecular identification.

### 3.2. PCR

The DNA was extracted from cultured isolates and fresh tissue after bead-beating (QIAamp^®^ DNA Mini Kit (Qiagen, Hilden, Germany), as well as from formalin-fixed, paraffin-embedded tissues (FPPE; Qiagen FPPE DNA kit) following the manufacturer’s recommendations. Sequencing the ITS1-5.8S-ITS2 region of DNA extracted from isolates, using ITS1 (5-TCCGTAGGTGAACCTGCGG-3) and ITS4 (5-TCCTCCGCTTATTGATATGC-3) primers was performed as previously described [9]. High sequence alignment (>99%) was found in Genbank Blast analysis with reference strains of *A. alternata* (GenBank Accession No MZ066723) [10].

The DNA eluates from tissues were tested for *Aspergillus* DNA using an in-house real-time pan-*Aspergillus* PCR assay, as previously described [11]. Furthermore, they were tested with the commercial multiplex real-time PCR assay, MucorGenius^®^ PN-700 version (PathoNostics, Maastricht, The Netherlands) according to the manufacturer’s instructions, using a Rotor-Gene Q (Qiagen) device. In all cases, the PCR assays yielded negative results. Subsequently, the FPPE DNA extract was used for ITS amplification, as described above. The obtained PCR product was sequenced and the Blast analysis indicated high homology (>99%) with reference strains of *A. alternata* (GenBank Accession No MZ066724) [10].

### 3.3. Antifungal Susceptibility Testing

In vitro antifungal susceptibility was determined following both the CLSI M38 [12] and the EUCAST E.DEF 9.3.2 [13] broth microdilution reference methodologies. The 48 h CLSI/EUCAST minimum inhibitory concentrations (complete visual growth inhibition) were 1/0.5 mg/L for amphotericin B, >8/>8 mg/L for flucytosine, >64/>64 mg/L for fluconazole, 0.5/0.5 mg/L for posaconazole, 1/>16 mg/L for itraconazole (trailing growth was found with EUCAST at concentrations 0.5–16 mg/L), 4/4 mg/L for voriconazole and 8/>8 mg/L for isavuconazole. Echinocandins’ minimal effective concentrations (MECs) were 0.125/0.06 mg/L for anidulafungin and 0.125/0.125 mg/L for micafungin. The isolate was stored (normal saline with 10% glycerol, −70 °C) in the culture collection of the Clinical Microbiology Laboratory’s Mycology Unit of the “Attikon” University hospital (Athens, Greece) as AUH1869.

## 4. Discussion

Herein we reported a rare case of acute necrotizing invasive FRS caused by *A. alternata,* the first case in Europe, in a patient with profound neutropenia secondary to underlying hematologic malignancy. An extensive diagnostic workup was performed in order to verify the diagnosis and exclude other fungal pathogens. The case is classified as acute because of the rapid onset (<4 weeks), invasive because the hyphae were seen within the vessels and necrotizing because of the extensive infarction-like necrosis. The chronic inflammation seen in histopathology may be due to other causes or to chronic FRS before the onset of hematological disease when AIFRS was developed. There are two commonly implicated fungal pathogens in AIFRS; *Aspergillus* tend to predominate in neutropenic patients, while Mucorales are mostly seen among patients with poorly controlled diabetes [1]. Nevertheless, in our case, their presence was excluded by performing pan-*Aspergillus* and pan-Mucormycetes PCRs on both of the fresh and FPPE tissue samples, which yielded negative results. The exclusion of the most common pathogens of AIFRS is important for accurate diagnosis and proper therapeutic management. As cultures may miss the causative agent of AIFRS, particularly when present together with other opportunists, a molecular diagnosis can provide accurate diagnosis. The *Alternaria* spp. are ubiquitous, pigmented (also known as dematiaceous or phaeoid) filamentous fungi, which are well-known soil saprophytes and plant pathogens that infrequently cause infection in humans. Our patient was prone to *Alternaria* AIFRS, given her profound neutropenia and her involvement in agricultural activities. Although the isolation and identification for the genus level of *Alternaria* is relatively easy, identification to species levels requires a molecular identification, as the morphological differences among the different species are subtle and not always present.

To date, 20 cases of AIFRS contributing to *Alternaria* spp. have been described between 1984 and 2008 (Table 2). Practically all of these cases were reported from the USA (19/20, one from Chile), with the majority (15/20; 75%) being reported as part of monocentric retrospective epidemiological studies [14,15,16]. The fact that AIFRS was not reported before in Europe may have to do with the differences in the ecological niche of *Alternaria* and the environmental fungal burden, in combination with differences in the prevalence of high-risk patients and in exposure to fungi (rural versus urban population). The median age of the patients was 25 (range 2–56) years and female patients accounted for 65% of the cases. Overall, 19/20 were hematology patients (35% acute myelogenous leukemia, 30% ALL) with severe neutropenia (8–41 days pre-infection), while one patient had acquired immunodeficiency syndrome-related immunosuppression. *A. alternata* was isolated in four cases [15,17,18]; in the remaining cases, the etiological agents were not identified to the species level. Amphotericin B was the most frequently used antifungal agent, either alone [16,17,18,19], or in combination with flucytosine [16], itraconazole [14] or voriconazole plus caspofungin [14], whereas a case of voriconazole monotherapy was described but the details on the clinical outcome were not provided [20]. In all of the cases, systemic antifungal therapy was combined with surgery, whereas some patients received granulocyte colony-stimulating factor and/or white blood cell transfusions. Despite stabilization or improvement of the AIFRS, 6/16 died 5 days to 8 months post-surgical debridement due to their underlying disease or other infections, without sign of AIFRS at autopsy.

Of note, our patient was on anidulafungin therapy (100 mg q24h) for 12 days at the time that the *A. alternata* was isolated. To date, there are limited published data available regarding the antifungal susceptibility profile of *Alternaria* spp. [21,22]. Although anidulafungin has been found to demonstrate the highest in vitro antifungal activity against *A. alternata* (MEC_50_ 0.008–0.03 mg/L), corroborating our findings, its clinical effectiveness in the treatment of *Alternaria* infections remains to be determined since it is unclear how the in vitro MEC correlates with the treatment outcome. Echinocandins are fungistatic, rather than fungicidal, against filamentous fungi and this might be translated into a lower clinical efficacy. In fact, Lafaurie et al. reported that all of the breakthrough invasive aspergillosis cases occurred in high-risk patients being empirically treated with caspofungin, as opposed to none of the amphotericin B recipients [23], while breakthrough invasive mold infections emerging during echinocandin prophylaxis were recorded in up to ~7.5% of the hematology patients [24]. Particularly for anidulafungin, a recent in vitro pharmacokinetic/pharmacodynamic study showed that the probability of target attainment for *Aspergillus fumigatus* isolates with the standard dose of 100 mg q24h was 0%, and remained low (10%) with the highest dose simulated (1500 mg q24h) even for isolates wit low MECs [25]. Furthermore, because of the small volume of distribution, tissue penetration is limited and therefore anidulafungin concentrations at the site of infection might have been subtherapeutic. Even in the best case scenario, where free drug levels in the blood are similar to the free drug levels in the tissue, those would not exceed 0.01 mg/L (based on total C_max_ of 7 mg/L and unbound fraction of 1%) which is below the MEC of the *Alternaria* isolate (0.125/0.06 mg/L). Therefore, clinicians should be aware of a possible breakthrough mold infection in neutropenic patients, especially those receiving echinocandins.

The optimal management of AIFRS requires a multidisciplinary approach that includes surgery, antifungal therapy and reversal of the underlying immunosuppression [4]. Considering that a delay in therapy of ≥6 days has been associated with a twofold increase in mortality [26], empiric antifungal treatment should be started as soon as possible when the clinical suspicion for AIFRS is high. The initiation of liposomal amphotericin B, transitioned to a targeted therapy when/if a fungal pathogen is identified, has been recommended in cases of immunocompromised cancer and transplant patients with AIFRS [27]. Based on the clinical breakpoints and epidemiological cut-off values established for *A. fumigatus* [28,29], our isolate was susceptible/wild-type to amphotericin B (CLSI/EUCAST MIC 1/0.5 mg/L), which is in line with previous findings [21,22]. The administration of liposomal amphotericin B was continued for a total of 38 days after *A. alternata* isolation. Currently, the treatment of alternariosis has not been standardized [30]. The ECMM-ESCMID guidelines consider itraconazole, voriconazole, posaconazole and amphotericin B as the cornerstones of the antifungal management of cutaneous and subcutaneous alternariosis [31]. Nevertheless, the previous reports on *Alternaria* AIFRS have shown that the combination of surgical debridement and antifungal monotherapy with amphotericin B contributes to a favorable clinical outcome [16,17,18,19].

In the present study, a case of breakthrough AIFRS by *A. alternata* with extensive infarction-like tissue necrosis, angioinvasion and severe chronic inflammation was described in an ALL patient receiving anidulafungin therapy. This is the first case of AIFRS by *A. alternata* in Europe. The patient was a farmer and was diagnosed with a hematological malignancy, both factors making her vulnerable to fungal infection. Prompt diagnosis, antifungal medication and surgical intervention are the cornerstone of successful treatment of AIFRS. Follow-up for several months is crucial for diagnosing possible recurrence.

**Table 2:** Overview of published acute invasive fungal rhinosinusitis cases due to *Alternaria* spp.

Author, Year of Publication (Reference)	Country, Study Period	Gender/Age (Years)	Predisposing Factors	Signs	Histopathology	Culture	Treatment	Outcome(Months Post-Surgery)
Morrison, 1993 [16]	USA, 1984	M/38	CML BMT (47 days pre-infection) Neutropenia	Bloody crust on nasal septum, necrotic appearing	Septate branching fungal hyphae invading the involved sinonasal tissue with areas of necrosis and vascular invasion	*Alternaria* spp.	Surgical debridement AMB therapy WBC transfusions	Died due to respiratory and graft failure; no sign of infection at autopsy (3 weeks)
Morrison, 1993 [16]	USA, 1985	M/25	ALL BMT (27 days pre-infection) Profound neutropenia	Superficial ulcer on nasal septum	Septate branching fungal hyphae invading the involved sinonasal tissue with areas of necrosis and vascular invasion	*Alternaria* spp.	Surgical debridement Antifungal therapy (AMB+5-FC) WBC transfusions	Alive (NA)
Morrison, 1993 [16]	USA, 1985	M/13	ALL BMT (15 days pre-infection) Profound neutropenia	Necrotic black lesion on nasal septum	Septate branching fungal hyphae invading the involved sinonasal tissue with areas of necrosis and vascular invasion	*Alternaria* spp.	Surgical debridement AMB therapy WBC transfusions	Alive (NA)
Morrison, 1993 [16]	USA, 1985	F/26	AML BMT (14 days pre-infection) Profound neutropenia	Small dark crust on middle turbinate	Septate branching fungal hyphae invading the involved sinonasal tissue with areas of necrosis and vascular invasion	*Alternaria* spp.	Surgical debridement AMB therapy	Alive (NA)
Morrison, 1993 [16]	USA, 1985	F/37	Aplastic anemia BMT (15 days pre-infection) Profound neutropenia	Black plaque on nasal septum	Septate branching fungal hyphae invading the involved sinonasal tissue with areas of necrosis and vascular invasion	*Alternaria* spp.	Surgical debridement AMB therapy	Died of aspiration pneumonia due to nasal bleeding; no sign of infection at autopsy (5 days post-surgery)
Wiest, 1993 [18]	USA, 1985	M/31	Acquired immunodeficiency syndrome	Black, necrotic-appearing lesion on the mucosa of the right nasal septum	Necrotic tissue with acute inflammation. Numerous, large, irregular septate hyphae	*A. alternata*	Surgical debridement AMB therapy	Died; no sign of infection at autopsy (8)
Morrison, 1993 [16]	USA, 1989	F/39	CLL BMT (27 days pre-infection)	Septal pallor, blood crusts in nose	Septate branching fungal hyphae invading the involved sinonasal tissue with areas of necrosis and vascular invasion	*Alternaria* spp.	Surgical debridement AMB therapy	Alive (NA)
Iwen, 1997 [19]	USA, 1985–1994	F/35	AML Profound neutropenia (30 days pre-infection)	Nasal necrosis	Pigmented, branching and septated moniliform hyphae	*Alternaria* spp.	Surgical debridement AMB therapy (25 days)	Alive (NA)
Chen, 2004 [17]	USA, 2003	F/56	AML Chemotherapy-induced neutropenia	Anterior nasal septal necrotic lesion with the overlying mucosa being slightly edematous and having a dusky appearance.	Multiple small fragments of squamous mucosa with ulceration and necrosis mixed with blood clot. Numerous hyphae elements with microconidia.	*A. alternata*	Surgical debridement AMB therapy	Died due to bacterial sepsis, but multiple cultures of nasal sinus content were negative (3)
Park, 2005 [15]	USA, 2000–2004	Pediatric patient	Hematologic malignancy Profound neutropenia	Necrosis	NA	*A. alternata*	Surgical debridement Antifungal therapy	NA
Park, 2005 [15]	USA, 2000–2004	Pediatric patient	Hematologic malignancy Profound neutropenia	Necrosis	NA	*A. alternata*	Surgical debridement Antifungal therapy	NA
Rabagliati, 2009 [20]	Chile, 2004–2008	Adult patient	Hematologic malignancy Chemotherapy-induced neutropenia	NA	NA	*Alternaria* spp.	Surgical debridement VRC therapy	NA
Montone, 2011 [32]	USA, NA	F/51	AML	NA	Invasion of fungal forms into submucosal tissue with frequent areas of tissue necrosis and angioinvasion	*Alternaria* spp.	NA	NA
Ardeshirpour, 2014 [14]	USA, 1994–2007	F/6	ALL Profound neutropenia (41 days pre-infection)	NA	NA	*Alternaria* spp.	Surgical debridement Antifungal therapy (AMB+CAS+VRC) G-CSF	Died from recurrent ALL, but without infection (2)
Ardeshirpour, 2014 [14]	USA, 1994–2007	F/11	AML Profound neutropenia (23 days pre-infection)	NA	NA	*Alternaria* spp.	Surgical debridement Antifungal therapy (AMB,+CAS+VRC) G-CSF	Alive (53)
Ardeshirpour, 2014 [14]	USA, 1994–2007	F/9	AML Profound neutropenia (13 days pre-infection)	NA	NA	*Alternaria* spp.	Surgical debridement Antifungal therapy (AMB+CAS+VRC) G-CSF	Alive (48)
Ardeshirpour, 2014 [14]	USA, 1994–2007	M/11	ALL Profound neutropenia (16 days pre-infection)	NA	NA	*Alternaria* spp.	Surgical debridement Antifungal therapy (AMB/L-AMB+ITC) G-CSF, WBC transfusions	Died from *Scopulariopsis* spp. throughout central nervous system (4)
Ardeshirpour, 2014 [14]	USA, 1994–2007	M/2	ALL Profound neutropenia (22 days pre-infection)	NA	NA	*Alternaria* spp.	Surgical debridement Antifungal therapy (AMB+ITC) G-CSF, WBC transfusions	Alive (32)
Ardeshirpour, 2014 [14]	USA, 1994–2007	F/4	AML Profound neutropenia (8 days pre-infection)	NA	NA	*Alternaria* spp.	Surgical debridement Antifungal therapy (AMB+ITC) WBC transfusions	Alive (37)
Ardeshirpour, 2014 [14]	USA, 1994–2007	F/13	ALL Profound neutropenia (16 days pre-infection)	NA	NA	*Alternaria* spp.	Surgical debridement Antifungal therapy (AMB+ITC) G-CSF, WBC transfusions	Alive (24)

M: male; F: female; ALL: acute lymphoblastic leukemia; AML: acute myeloid leukemia; CLL: chronic lymphocytic leukemia; CML: chronic myelogenous leukemia; BMT: bone marrow transplantation; WBC: white blood cell; G-CSF: granulocyte colony-stimulating factor; AMB: amphotericin B, 5-FC: flucytosine; ITC: itraconazole; VRC: voriconazole; L-AMB: liposomal amphotericin B; CAS: caspofungin; NA: not available.

## Figures and Tables

**Table 1:** Laboratory parameters on the day of acute lymphoblastic leukemia (ALL) and fungal rhinosinusitis (FRS) diagnosis.

Laboratory Parameters	On the Day of ALL Diagnosis (22/6)	On the Day of FRS Diagnosis (14/7)
CRP (mg/L)	332	88.8
Glucose (mg/dL)	105	119
Urea (mg/dL)	35.3	22.6
White Blood Cells (10^9^/L)	24.8	0.33
Neutrophils (10^9^/L)	0.23	0.16
Lymphocytes (10^9^/L)	18.30	0.16
Monocytes (10^9^/L)	6.07	0.01
Basophils (10^9^/L)	0.15	0
Red Blood Cells (10^12^/L)	3.5	2.67
Hemoglobin (g/L)	10.3	7.9
Hematocrit (%)	29.8	22.4
Platelet (10^3^/μL)	70	56

## Data Availability

Data available on request.
